# Detection Analysis of Epileptic EEG Using a Novel Random Forest Model Combined With Grid Search Optimization

**DOI:** 10.3389/fnhum.2019.00052

**Published:** 2019-02-21

**Authors:** Xiashuang Wang, Guanghong Gong, Ni Li, Shi Qiu

**Affiliations:** ^1^State Key Laboratory of Virtual Reality Technology and Systems, Beihang University, Beijing, China; ^2^Automation Science and Electrical Engineering, Beihang University, Beijing, China; ^3^Xi'an Institute of Optics and Precision Mechanics, Chinese Academy of Sciences, Xi'an, China

**Keywords:** continuous electroencephalography, grid search optimization, random forest, epileptic seizure detection, simulation model

## Abstract

In the automatic detection of epileptic seizures, the monitoring of critically ill patients with time varying EEG signals is an essential procedure in intensive care units. There is an increasing interest in using EEG analysis to detect seizure, and in this study we aim to get a better understanding of how to visualize the information in the EEG time-frequency feature, and design and train a novel random forest algorithm for EEG decoding, especially for multiple-levels of illness. Here, we propose an automatic detection framework for epileptic seizure based on multiple time-frequency analysis approaches; it involves a novel random forest model combined with grid search optimization. The short-time Fourier transformation visualizes seizure features after normalization. The dimensionality of features is reduced through principal component analysis before feeding them into the classification model. The training parameters are optimized using grid search optimization to improve detection performance and diagnostic accuracy by in the recognition of three different levels epileptic of conditions (healthy subjects, seizure-free intervals, seizure activity). Our proposed model was used to classify 500 samples of raw EEG data, and multiple cross-validations were adopted to boost the modeling accuracy. Experimental results were evaluated by an accuracy, a confusion matrix, a receiver operating characteristic curve, and an area under the curve. The evaluations indicated that our model achieved the more effective classification than some previous typical methods. Such a scheme for computer-assisted clinical diagnosis of seizures has a potential guiding significance, which not only relieves the suffering of patient with epilepsy to improve quality of life, but also helps neurologists reduce their workload.

## Introduction

Epilepsy is the clinical manifestation of hyperpolarizing electrical activity in paroxysmal neurons in the brain, which has recurrent, sudden, and transient characteristics (Patidar and Panigrahi, 2017). Electroencephalography (EEG) was introduced by Berger (1929) to measure electrical activity in the brain. One of the main applications of EEG in clinical diagnostics is the automatic detection of epileptic seizures (Navarro et al., 2003; Loui et al., 2014). Continuous electroencephalography (cEEG) (Wang et al., 2018) is a monitoring tool for epileptic seizures in the intensive care unit (ICU). Other physiological detection methods cannot reflect seizure information in real time in the manner of cEEG. However, epileptic seizure signals cannot be interpreted in the short term, and it is therefore essential to record EEG signals continuously over a long period, which typically involves many hours of recording the patient's brain waves (Kennedy and Gerard, 2002; Gavvala et al., 2015). Clinically, due to the subtle signs of seizure of epileptic patients, only 35% of non-convulsive seizures (NCS) and non-convulsive status epilepticus (NCSE) can be diagnosed by neuro-medical doctors although 52% of patients have disease episode in the ICU ward (Claassen et al., 2004; Scheuer, 2010). The probability of detecting morbidity is so low. It is still necessary to perform manual visual detection and identification by experienced neurologists or neuro-electrophysiologists. Since the introduction of cEEG detection technology into the monitoring of the ICU ward, the recognized results have significantly improved the diagnostic rate of doctors while assisting doctors to make decisions (Abend et al., 2011).

Therefore, there is an urgent requirement for an automated framework to detect and recognize seizures, to enable efficient treatment to be administered quickly. This requires is a highly classified accurate (ACC) and efficient automatic detection algorithm including time domain, frequency domain, time-frequency domain and non-linearity analysis, to allow long term monitoring, and seizure detection. In general, seizure detection involves two steps in feature extraction and feature classification with important attributes of the cEEG being extracted in the feature extraction step and provided to the recognizer as inputs.

In recent years, several studies solved automatic monitoring and recognizing problem to non-stationary EEG at the onset of epilepsy. A classic example is the Welch spectral analysis method introduced into the feature analysis of epileptic seizure detection. Tzallas et al. used the time domain method through computerized EEG analysis during epileptic seizures (Liu et al., 1992). Additionally, a popular Fourier-based technique for spectral analysis has commonly been to analyze EEG signals in the frequency domain. Polat et al. proposed a hybrid model for seizure detection using a fast Fourier transformation and decision tree for feature extraction and classification, respectively (Polat and Güne, 2007). A flexible wavelet transformation and the fractal dimension of the time-frequency method also have been used for seizure segment detection in long-term EEG (Li et al., 2016; Satapathy et al., 2016; Swami et al., 2016; Sharma et al., 2017).

Independent components analysis (ICA) (Whitmer et al., 2010) and linear discriminant analysis have been reported for EEG signal extraction and classification (Subasi and Ismail Gursoy, 2010). Lately, a multiscale radial basis function algorithm showed promising results in the decoding of EEG of epileptic seizures (Li et al., 2017). The following content briefly discusses the widely used time-frequency methods of fast Fourier transform (FFT), wavelet transform (WT), ICA and power spectral density (PSD), which have all been applied to the time frequency domain for the detection of epileptic activity (Qinghua et al., 2003; Tzallas et al., 2009; Boashash et al., 2012; Wang et al., 2018).

After considering the above literature, we considered the FFT transform to have several disadvantages for time-frequency analysis. First, the FFT transform cannot do a good job of solving the EEG analysis problem using a fixed window function. Second, it is very time-consuming. In this study, we adopted a short time Fourier transform (STFT) to conduct the time-frequency analysis of non-stationary EEG signals by adjusting different time windows to avoid the disadvantages of the FFT transform. The mean energy, standard deviation, and high amplitude gamma frequency of signals processed by the STFT approach are formed the feature vectors for the recognition model.

After obtaining appropriate features, the final step is to feed these features into a suitable classifier. Numerous machining learning models have been developed for seizure detection (Guerrero-Mosquera et al., 2010; Parvez and Paul, 2014; Jaiswal and Banka, 2018; Sharma and Pachori, 2018), with the classification methods of empirical mode decomposition (EMD), principle component analysis (PCA), and genetic algorithms (GA) having been proposed. Support vector machine (SVM) of learning was adopted by Boser et al. (1992) and Kai Fu et al. (2015), while He et al.'s neural network (NN) classified technique were used in the early days of machine learning applied to the field of brain science (He et al., 2004, 2006). Zhang et al.'s artificial neural network (ANN) technique achieve 89% classification accuracy (Zhang et al., 2007). Brabanter et al. proposed a least squares support vector machine (LS-SVM) of the best pattern classification approach. Their LS-SVM technique was used for the classification of two-level of seizure and non-seizure EEG signals from the small seizure dataset of Bonn University. They obtained 98.0–99.5% accuracy using a radial basis function (RBF) kernel, and 99.5–100% accuracy using a Morlet kernel (Brabanter et al., 2010). Yang Li's analysis technique used a K-Nearest Neighbors (K-NN) algorithm for epileptic seizure detection with classification based on the EEG signals. The classification results indicated that it can achieve a high classification accuracy of 99.1% (Wang et al., 2017).

In the above-mentioned literature, the algorithms classify two types, which are EEG data into two types, seizure EEG epochs and non-seizure EEG epochs. Such a two-way classification of the EEG is unfavorable in practical applications, as in reality there are multiple degrees of epileptic seizure. Therefore, our random forest-grid search optimization (RF-GSO) model classifies the cEEG dataset work to build classification model of three categories to cEEG dataset into three categories representing non-epilepsy, severe epilepsy, and intermittent epilepsy. This classification not only saves the time for the two-two classification between three datasets but also effectively avoids the misdiagnosis or missed diagnosis caused by manual analysis and recognition. Our model can classify three levels of epilepsy in one go. At the same time, we can also classify the dataset by classifying the model.

Therefore, we believe our RF-GSO classification algorithm has the following advantages for training on epilepsy EEG:

a) The random forest classification algorithm has a high degree of parallelization, which improves operational efficiency. This is advantages for training on large quantities of EEG data. However, it does generate a large number of hyper-parameters during the training process, and it may be difficult to determine the optimal parameters. This is where the GSO optimization algorithm is useful, as it can accelerate the search for the optimal combination of parameters by filtering them repeatedly with a variable step size, thus generating an optimal RF model.
b) As the decision tree node-partitioning features can be selected randomly, the model can still be effectively trained when the sample feature dimension is high. By using GSO and 10-fold cross validation (CV), the situation where an excessively high penalty function causes an over-learned state to occur can be avoided. The implementation of 10-fold CV on the training set increases the robustness and adaptability of the trained RF classifier.
c) The trained optimized RF classifier can predict the three types of conditions for different degrees of patients. At the same time, the classifier is malleable and can be used to solve the multiple degrees of disease prediction in patients with epilepsy by modifying the model, which doesn't limited to the classification of the two conditions.
d) In this study, the time-frequency analysis method is used to extract the time-frequency characteristics of EEG signals. At the same time, the statistical characteristics of EEG signals are extracted by statistical techniques, thus the best combination of feature extraction and feature classification is realized.
e) The RF model is not sensitive to partial feature deletions. Therefore, the classification results are robust to dimension reduction processing by PCA, which is especially relevant for cEEG. Our model can predict several thousand explanatory variables effectively.

**Figure 1:**
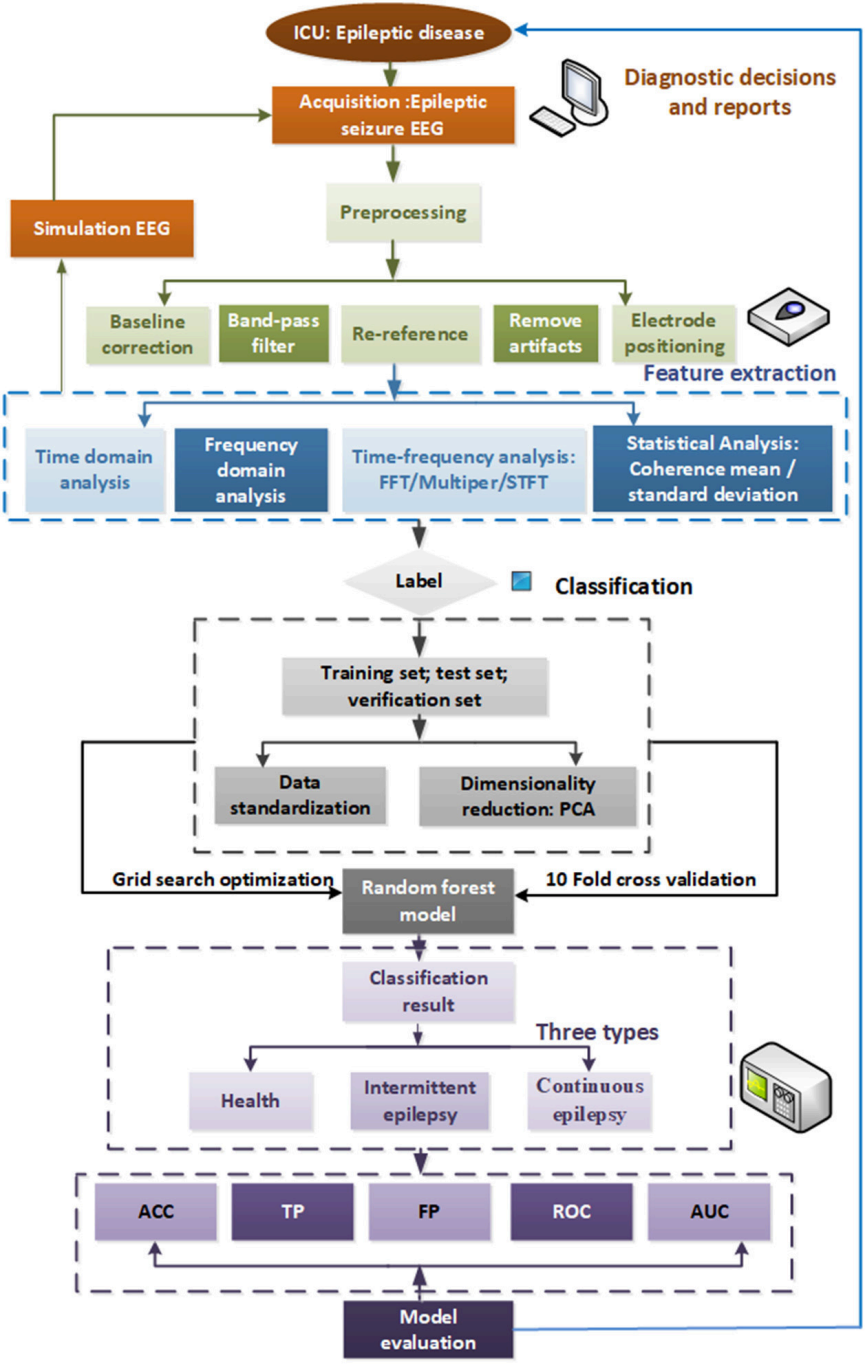
Automatic detection framework for seizure EEG.

The automatic detection framework for epileptic seizure EEG studied in this paper is illustrated in Figure 1. First, we preprocess the collected medical cEEG data, then we perform T-F analysis on the simulated and real data. The frequency features of frequency and statistics of the EEG signals are extracted by STFT, and the reduced features are fed into the random forest model. We use the GSO optimization algorithm to optimize the RF hyper-parameters to achieve a better model. Finally, we conduct multi-indexes assessment of our model's ability to detect the three levels of seizure status.

The remainder of the paper is organized as follows. In section Dataset and Feature Extraction Methods, we apply the different T-F analysis methods to real EEG data after first preprocessing it. Comparisons of the results of the different approaches revealed that STFT attained the best effects. The study adopts the PCA method to reduce the dimensionality of the cEEG features. In section Random Forest Algorithm Based Grid Search Optimization, we feed the extracted features into our novel automatic detection model using 10-fold CV to obtain the three classification categories of seizure, light-seizure and non-seizure. In section Experimental Results and Discussions, the experimental results are analyzed using ACC, confusion matrix, receiver operating characteristic curve (ROC), and area under curve (AUC) generated by sensitivity, and specificity. Finally, our contributions are summarized and our future work is discussed in section References.

## Dataset and Feature Extraction Methods

We establish a mathematical simulation model of perfect EEG signals, and use different feature extraction methods to analyze the EEG signals while ensuring the credibility of the simulation. The obtained results are consistent with the conditions of the modeling hypothesis indicating that the method has applicability to the processing of real clinical EEG data.

### Simulated EEG Data Model

**Figure 2:**
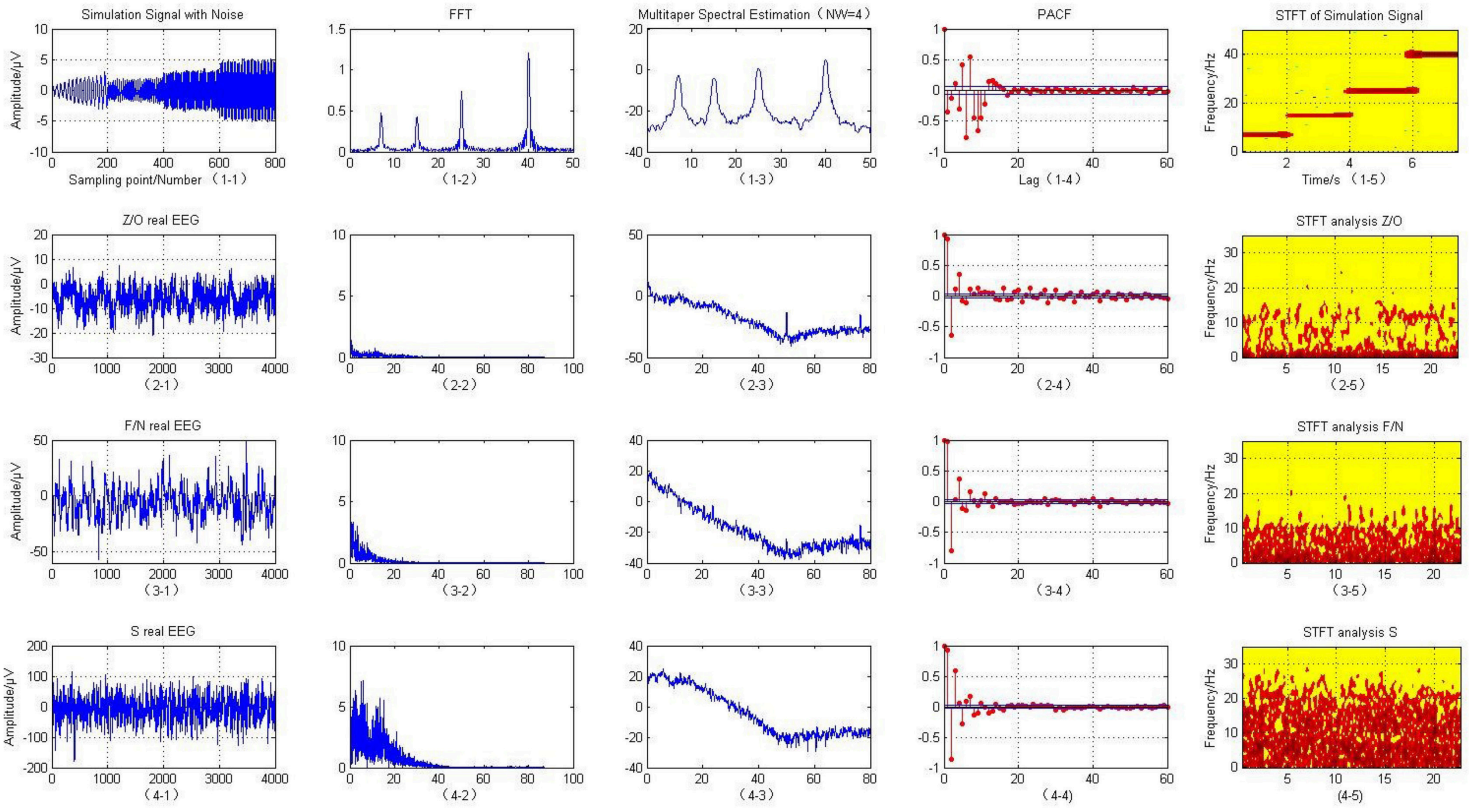
T-F analysis of simulated and actual clinical EEG data.

First, the four frequency sub-bands including theta (4–8 Hz), alpha (8–16 Hz), beta (16–32 Hz), and gamma (32–60 Hz) (Trenado, 2015; Amiri et al., 2016) were created for the simulated EEG model. These signals were created with a sampling frequency of 100 Hz, and corrupted by the addition of Gaussian white noise with a sequence of variance of 0.04, as shown in Figure 2 (1-1). The simulation modeling is defined as follows:

(1) $$\begin{array}{c} y\left(t\right)=\{\begin{array}{c} 2{\left|t\right|}^{\omega }\sin\left(2\pi f_{\theta }t\right),t\in \left[0,2\right);\\ 2{\left|t\right|}^{\omega }\sin\left(2\pi f_{\beta }t\right),t\in \left[2,4\right);\\ 2{\left|t\right|}^{\sigma }\sin\left(2\pi f_{\alpha }t\right),t\in \left[4,6\right);\\ 2{\left|t\right|}^{\sigma }\sin\left(2\pi f_{\gamma }t\right),t\in \left[6,8\right);\\ 0,otherwise \end{array} \end{array}$$

Using the simulated EEG expression, we can clearly see four peaks and lines on the spectrum representing the four frequency components of 7, 15, 25, and 40 Hz when analyzing it with the time-frequency approaches of FFT, multitaper spectrum and STFT, as shown in the first row of Figure 2. These results confirm that the original time domain signals mainly contained these four different frequency signals. They are also the point of energy concentration, which is consistent with the simulating signals. Some small peaks other than these four peaks are present when using the FFT, these are due to spectral leakage, which may cause the spectrum to be blurred and distorted. However, the STFT algorithm clearly shows four red lines representing the four artificial EEG signals, which verifies that this technique captures the most detailed frequency information of the model.

### Real Clinical EEG Data Analysis

Non-invasive EEG data was obtained at Bonn University from 25 patients with medically intractable partial epilepsy. These consecutive patients were selected according to the following inclusion criteria.

The datasets were divided into five groups of ictal scalp EEG signals: O, Z, F, N, and S. Each group of data contained a total of 100 samples from 5 subjects. The raw EEG data was recorded using a standard 10–20 system with a sampling frequency of 173. 61 Hz. The age of the subjects ranged from 19 to 60 years, they were all right-handed, and the locations of the epileptogenic foci for each subject were identified by experienced epileptologists. More detailed information on the five cEEG datasets is provided in Table 1.

**Table 1:** Description of real EEG data.

**Dataset**	**Subject condition**	**Epileptogenic foci**	**Electrode collection area**	**Subject status**	**Samples Number**
O/Z	health	Scalp surface	All brain areas	All areas	200
F/N	seizure-free intervals	Intracranial	Lesion outside/inside area	intermission	200
S	seizure activity	Intracranial	Intralesional area	Attack period	100

This paper uses the O/Z, F/N, and S datasets to classify the EEG signals. The O/Z datasets were from healthy subjects under a state of alert and only used EEG signals acquired from the surface of the scalp. The F/N datasets were from epilepsy patients who did not suffer a seizure within the area covered by the intracranial EEG signals during the data acquisition period. The S dataset was from patients with seizure activity that caused lesions within the area of the intracranial EEG. The raw EEG are expressed in the first column of the matrix in Figure 2.

### EEG Preprocessing and Time-Frequency Analysis

#### EEG Preprocessing

EEGLAB toolbox of Matlab was used to preprocess the cEEG. This software allows preprocessing of the raw EEG signals, including adding electrode channel positions, digital filtering, removing artifacts, re-referencing, and baseline corrections.

EEG is mainly distributed in the frequency range of 0–40 Hz. Therefore, a Butterworth digital low-pass filter (Yan and Yuan, 2017) with a cut off frequency of 40 Hz was used to extract the effective frequency band. The absolute value of the data is taken in the experiment to avoid negative energy. To ensure the credibility of the test results, the arithmetic average processing was performed for the above three groups of data and they were compressed into single column matrices. The energy mean, number of cases, and variance of the F/N, O/Z, and S datasets are shown in the Table 2. It can be observe that the S set had the largest standard deviation and the highest mean energy.

**Table 2:** Statistic feature of real EEG data.

	**FN**	**OZ**	**S**
Mean	−5.94	−6.31	−4.74
Number of cases	4,097	4,097	4,097
Standard deviation	13.10	4.56	38.55

#### Time-Frequency Analysis

Fourier transform, multitaper spectral analysis, PACF, and STFT were then used to describe the time-frequency features of the signals. Figure 2 (2-2, 3-2, 4-2) illustrates that the Fourier transform is an analysis of the frequency of the entire cEEG signals; to a certain degree, it reflects the frequency characteristics of the entire signals. Therefore, the non-stationary signals or frequency change slowly with time. It is possible to smooth signals using an FFT; however, for EEG signals, whose frequency changes rapidly with time, the fast changing frequency is effectively “averaged” with the FFT, and it can only give the overall effect of the signals, it cannot reflect the frequency variation characteristics of the signals themselves. With the STFT, a window is added to the signals and this window function is moved. Assuming that the windowed signals represent stationary signals in different finite time widths, the power spectrum at different moments can be calculated. STFT treats non-stationary brain signals as stationary signals and superimposes a series of short signals. It performs windowing to obtain a two-dimensional function of three types of brain electrical signals: Z/O, F/N, and S. Their corresponding time–frequency energy distributions are shown in Figure 2. Compared with the FFT results, the spectra of the three data sets Z/O, F/N, and S are significantly different, and the analysis results using the STFT are clearer. Compared with the Z/O and F/N data sets Figure 2 (2-5, 3-5), the EEG signals in the S dataset have much higher energy, as shown in Figure 2 (4-5). Wave peaks are present in the alpha and theta bands, especially in the S dataset, with high energy near 0–3 and 7 Hz frequencies. The overall energy in the F/N datasets was lower, with mainly slow wave activity with low amplitude and wave peaks that were not particularly obvious. The brain power of the Z/O subjects was the lowest of the datasets. Therefore, it can be inferred that the brain power of the S datasets represents severe and persistent epileptic episodes, as seen in Figure 2 (4-5). The brain power of the F/N datasets represents mildly epileptic patients with latent epilepsy. In the Z/O dataset, the EEG spectrum showed a little energy concentration, and the Z/O datasets are the EEG data of healthy people, as in Figure 2 (2-6).

### Analysis Features for the Epilepsy Monitoring Model

The cEEG scalp signals at time *t* can be defined as a vector:

(2) $$\begin{array}{l} \text{S}\left(t\right)=\left(\text{s}\left(t_{1}\right),\text{ s}\left(t_{2}\right),\cdots \;,\text{s}\left(t_{n}\right)\right)=\left(\begin{array}{lll} s_{11} & \cdots & s_{1n}\\ \vdots & \ddots & \vdots \\ s_{p1} & \cdots & s_{pn} \end{array}\right) \end{array}$$

S(t) (*n* = 1, 2, … , 4097) represents the cEEG signals. For each segment of the EEG signals, an MVAR model with p order can be built as follows:

(3) $$\begin{array}{l} \text{S}\left(t\right)=\sum_{i=1}^{p}\omega_{i}S\left(t-i\right)+\varepsilon \left(t\right) \end{array}$$

ω_*i*_ is a matrix of coefficients and ε(*t*)is the estimation error which is a time sequence of Gaussian white noise (Wei and Yan, 2017). The order *p* can be depended by the Schwarz's Bayesian Criterion. The 4 order of the MVAR model can describe the features of the cEEG signals. Therefore, the STFT of coefficient ω_*i*_ can be calculated by:

(4) $$\begin{array}{l} \omega \left(\text{f}\right)=\sum_{i=1}^{p}\omega_{i}e^{-2\pi irf} \end{array}$$

The leaf transformation then moves the window function to repeat the above calculations for different moments. The expression is as follows:

(5) $$\begin{array}{l} T_{s}\left(t,f\right)=\int_{-\infty }^{+\infty }{s\left(\tau \right)k}^{*}\left(\tau -t\right)e^{-jf\tau }d\tau \end{array}$$

To optimize the performance of the algorithm and improve the accuracy of the classification, standardized transformation of the data was performed. At the same time, PCA was used for feature extraction and dimension reduction, to allow the relevant important features in the original data to be more easily revealed.

(6) $$\begin{array}{l} S_{i}^{\prime }*S_{j}^{\prime }=0,\;\left(\text{i}\neq \text{j}\right) \end{array}$$

The average frequency, standard deviation, and average amplitude of the EEG signal are extracted as features for identification, to form a three-dimensional observation vector. Among them, the average frequency is:

(7) $$\begin{array}{l} \text{P}=\frac{\sum f_{j}M_{j}}{\sum M_{j}} \end{array}$$

where *M*_*j*_ is the power spectrum value of the frequency. It is very important to choose the right model type and order for power spectrum estimation based on the parametric model; otherwise, it may cause large errors in the results.

The time-frequency analysis reveals the frequency distribution of the signal and the regularity of each frequency component over time. The principle of STFT is to use a window function *k*(τ − *t*) to extract a section of EEG centered on a certain moment and to then perform a Fourier transform on the section.

In the PCA algorithm (Frances and Robert, 2013), the EEG signal data is converted from the original coordinate system to a new coordinate system, and the selection of this new coordinate system is determined by the data itself, because the maximum variance of the data provides the most important information. When converting the coordinate system, the direction with the largest variance is used as the coordinate axis direction. The first new coordinate axis selects the method with the largest variance $S_{i}^{\prime }$ in the original data, and the second new coordinate axis selects the orthogonality with the first new coordinate axis and the direction of the second largest variances $S_{j}^{\prime }$. This process is repeated, a number of times, and is the feature dimension of the original data.

In the new coordinate system obtained in this way, most of the variances are contained in the first few axes, with the subsequent axes containing a variance of almost zero. This method retains dimensional features that contain most of the variance and ignores feature dimensions that contain variances of almost zero, thereby achieving dimensionality reduction of the data features.

The PCA Algorithm 1 is described below:

**Algorithm 1:** Principal component analysis (PCA)

	**Input**: Sample set $\text{S}\left(t\right)={\left(\text{s}\left(t_{1}\right),\text{ s}\left(t_{2}\right),\cdots \;,\text{s}\left(t_{n}\right)\right)}^{T}$ Low dimensional dimension *n*, **Process**:
1:	Centralize all samples:
	$$S\left(t_{i}\right)\leftarrow S\left(t_{i}\right)-\frac{1}{n}\sum_{i=1}^{n}S\left(t_{i}\right)$$
2:	Calculate the covariance matrix of sample: *SS*^*T*^
3:	Solving the correlation coefficient matrix
	$$R={\left(r_{ij}\right)}_{n\times n}\left(r_{ij}=r_{ji},r_{ii}=1\right)$$
4:	Solving the eigenvalues of the correlation coefficient matrix:
	$$\lambda_{1}\geq \lambda_{2}\cdots \geq \lambda_{n}\geq 0$$
5:	Determine the number of principal components: m
	$$\overset{m}{\underset{i=1}{\sum }}\lambda_{i}/\overset{p}{\underset{i=1}{\sum }}\lambda_{i}\geq \alpha ,\;\alpha =80\%$$
6:	Calculate the corresponding eigenvector:
	$$x_{1}=\left(\begin{array}{c} x_{11}\\ x_{21}\\ \vdots \\ x_{p1} \end{array}\right),x_{2}=\left(\begin{array}{c} x_{12}\\ x_{22}\\ \vdots \\ x_{p2} \end{array}\right),\ldots ,x_{m}=\left(\begin{array}{c} x_{1m}\\ x_{2m}\\ \vdots \\ x_{pm} \end{array}\right)$$
7:	Calculate principal components:
	$$Z_{i}=x_{1i}S_{1}+x_{2i}S_{2}+\cdots +x_{pi}S_{p}$$
	i=(1,2,⋯,m)

## Random Forest Algorithm Based Grid Search Optimization

### RF-GSO for the Machine Learning Model

The Random forest (RF) (Archer and Kimes, 2008) is an effective integrated machine learning method combined by decision trees. The RF identification method is suitable for high-dimensional data and runs fast. However, a large number of hyper-parameters are generated during the operation, and in order to obtain a higher accuracy of recognizing the epileptic EEG signal, the model parameters need to be optimized. At present, there are relatively few methods for optimizing the parameters in the random forest method, and it is usually based on experience to select manual parameters. In particular, the number of decision trees in the random forest method has a large influence on the performance of the model, and for different categories of data, the number of decision trees is different when the performance of the model is optimal. The parameters of the random forest method are selected only by experience, and the random forest identification model with the best performance is usually not obtained. This paper uses the improved GSO to identify RF by computer. The parameters are optimized, and the cross-validation method in machine learning is used to more effectively avoid the over-fitting problem of the trained random forest model.

Data distribution was performed on the data set, including test set, training set, and verification set. At present, the literature was divided into two groups for this dataset. In this study, the dataset was directly divided into the three categories of healthy people, intermittent epilepsy patients, and continuous epilepsy patients, which were labeled by “−1,” “1,” “0.”

The RF is an extended variant of Bagging. First, a bootstrap sample Z^*^ was randomly selected from the training set in a returning way. Taking the randomly selected data in the above steps as the training data, decision trees *T*_*b*_ were established. Second, a subset of M features is randomly selected from the feature set of each node of the decision tree. The RF tree is grown to enhance the binding data by recursively repeating the above steps for each terminal node of the decision tree until the decision tree can accurately identify the training data set while achieving the minimum node size. In the process of model training, this paper uses the recognition regression tree CART algorithm to split the nodes, and the Gini value of the Gini index is used as the basis of the splitting node. The sample training set Z^*^ contains different characteristics, and the Gini index of this training set is:

(8) $$\begin{array}{l} GINI\left(\text{k}\right)=1-\overset{k}{\underset{i=1}{\sum }}p_{i} \end{array}$$

where, *p*_*i*_ is the probability of a category *i* feature. The number of features corresponding to the sample training set were {*n*_1_, *n*_2_, … , *n*_*k*_}, *n* = *n*_1_+*n*_2_+*n*_3_, the split Gini index is:

(9) $$\begin{array}{l} GINI\left(M^{*}\right)=\frac{n_{1}}{n}GINI\left(M_{1}\right)+\frac{n_{2}}{n}GINI\left(M_{2}\right)+\frac{n_{3}}{n}GINI\left(M_{3}\right) \end{array}$$

Third, all decision trees ${\left\{T_{b}\right\}}_{1}^{m}$ are aggregated. For an input sample, the decision trees of *m* have recognition results of *m*, and the RF model inherits all the recognition voting results. Forecasting is performed on the new node, and the most recognized number of votes is the output $\{{\overset{\wedge }{C}}_{b}\left(x\right){\}}_{1}^{m}$. Pseudo code for the RF-GSO is shown in Algorithm 2.

**Algorithm 2:** Random Forest for Classification. (*RF, Z*_*i*_), GSO

1:	**For** *i* = 1 to m:
	(a) Draw a bootstrap sample Z^*^of size *P*from the training data. (b) Grow a random forest tree *T*_*b*_to the boost strapped data, by recursively repeating the following steps for each terminal node of the tree, until the minimum node size *n*_min_is reached.
2:	**Output** ensemble of tree ${\left\{T_{b}\right\}}_{1}^{m}$ To make a prediction at a new point *x* Classification: Let ${\overset{\wedge }{C}}_{b}\left(x\right)$ be the class prediction of the random forest tree. Then $\overset{\wedge }{C_{rf}^{m}}\left(x\right)=$ majority vote $\{{\overset{\wedge }{C}}_{b}\left(x\right){\}}_{1}^{m}$
3:	First coarse search hyper-parameters: penalty parameter, min_sample_leaf, max_features, n_estimators. step size:10 Second accurate search: reduce step size, st. min (penalty parameter) is the best group of parameters, step size:0.1.

The random forest identification algorithm generates a large number of hyper-parameters during the training process. It is difficult to calculate the optimal parameters of the recognition model by relying on experienced programmers to manually debug these generated parameters. In order to improve the classification performance of the random forest algorithm, this paper proposes an improved grid search algorithm to optimize and configure the parameters of the RF model.

**Figure 3:**
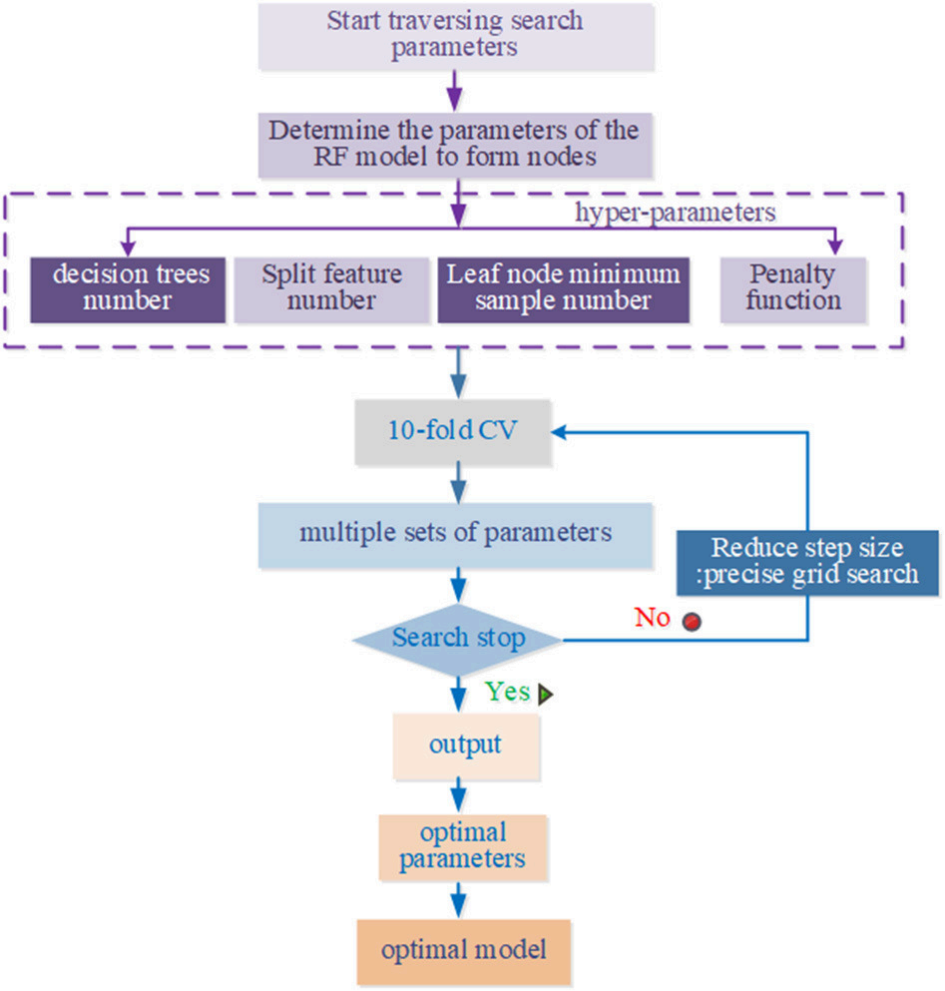
Parameters optimization flow to GSO.

The GSO algorithm refers to meshing the variable regions, then traversing all the grid points, solving the objective function values satisfying the constraints, and selecting the optimal values. It takes a lot of training time to traverse all the parameters on the grid. In this paper, the improved GSO algorithm is to improve the training speed. Specific steps are as follows:

First, we used a long distance step size for a rough search over a large range. Second, the mesh was built on the coordinate system, and its mesh nodes were the corresponding parameters pair of penalty parameters, the number of decision trees, the number of split features, min_sample_leaf, max_features, and n_estimators. The optimal parameters and recognition accuracy were output when there was a set of parameters that meet the requirements; we select the parameter with the smallest penalty parameter as a more selective object when multiple sets of parameters meet the requirements. Next, a second accurate search is performed in small steps on the set of parameters; repeat the above steps with the step set to 0.1 to find the global optimal hyper-parameters. The above parameter optimization flowchart of RF model based on the improved grid search algorithm is shown in Figure 3.

### K-fold Cross Validation

To reduce the influence of the selected training data and test data on the model evaluation, k-fold cross validation was used. This involves the training data being divided into subsets without repetition.

(10) $$\begin{array}{l} \left\{V_{1},V_{2},\cdots \;,V_{k}\right\},\left(V_{i}\cap V_{j}=\emptyset \right) \end{array}$$

k-1 subsets were used for training, and the remaining subset was used for testing. This process was repeated k times to obtain k accuracy values, which were averaged to provide a mean value for the evaluation. In past literatures, automatic seizure detection of L. Guo et al. Nicolaou et al. and Samiee et al. have no use the CV as seen from the following Table 3. Jianfeng Qu et al used the default 5-fold CV. In this study, 10-fold CV was used to obtain more reliable and robust performance results. The training set were randomly divided into ten subsets, with only one subset being used as the verification set. The other residual subsets were used to train the cEEG classifier on data corresponding to different levels of epileptic seizure. The use of 10-fold CV reduces the over-fitting phenomenon and increases the credibility of the data classification. The pseudo code for the 10-fold cross-validation is shown in Algorithm 3.

**Table 3:** Comparison of the main relevant previous research studies.

**References**	**Techniques**	**10-fold CV**	**Dataset**	**ACC%**
Guo et al., 2010	DWT and line length, ANN	no	{Z}-{S}	100
			{FNOZ}-{S}	97.7
Gandhi et al., 2011	DWT, energy and std, SVM, NN	yes	{FNOZ}-{S}	95.4
Nicolaou and Georgiou, 2012	Permutation entropy, SVM	no	{Z}-{S}	93.5
			{O}-{S}	82.8
			{N}-{S}	88.0
			{F}-{S}	79.94
			{FNOZ}-{S}	86.1
Alam and Bhuiyan, 2013	EMD, higher order moments, ANN	no	{O}-{S}	100
			{F}-{S}	100
			**{FN}-{OZ}-{S}**	**80**
Samiee et al., 2015	Rational short time Fourier	no	{Z}-{S}	99.8
			{O}-{S}	99.3
			{N}-{S}	98.5
			{F}-{S}	94.9
			{FNOZ}-{S}	98.1
Swami et al., 2016	DTCWT, energy an std, Shannon entropy features, RNN	yes	{Z}-{S}	100
			{O}-{S}	98.89
			{N}-{S}	98.72
			{F}-{S}	93.3
			{ZO}-{S}	99.1
			{NF}-{S}	95.1
			{FNOZ}-{S}	95.2
Sharma et al., 2017	ATFFWT and FD, LS-SVM	yes	{Z}-{S}	100
			{O}-{S}	100
			{N}-{S}	99
			{F}-{S}	98.5
			{ZO}-{S}	100
			{NF}-{S}	98.6
			{ZO}-{NF}	92.5
			{FNOZ}-{S}	99.2
Yuanfa Wang et al., 2018	DWT, SVM	no	**{FN}-{OZ}-{S}**	**93.9**
This work	STFT, mean energy std and PCA, RF and GSO	yes	{Z}-{S}	100
			{O}-{S}	100
			{N}-{S}	98.5
			{F}-{S}	98.1
			{ZO}-{S}	100
			{NF}-{S}	98.2
			{ZO}-{NF}	93.2
			{FNOZ}-{S}	98.5
			**{FN}-{OZ}-{S}**	**96.7**

**Algorithm 3:**
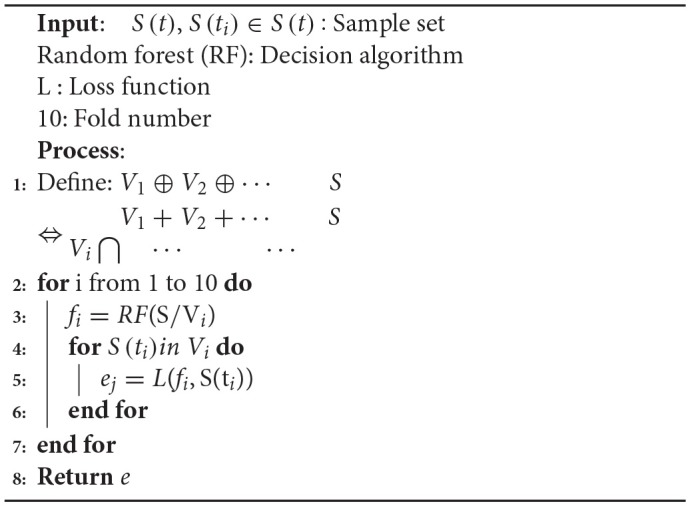
10-Fold Cross-validation(S, RF, L, 10)

## Experimental Results and Discussions

The experiments were performed on an Acer PC with a 2.8 GHz Intel Core i5-6200U CPU, 8 GB low voltage memory, 1 TB of storage, and a 64-bit operating system.

### Effects of RF Algorithm With Grid Search Optimization

The classification performance of the proposed RF algorithm should be determined on the statistical results of all patients, to avoid possible deviations from the detection results for epileptic seizures from a single patient. The identification and decision-making problem in the classification of epilepsy EEG data is also an unbalanced classification problem. The average and standard deviation of the performance per patient allow for statistical testing, to determine differences between the three categories, including healthy people and intermittent epilepsy patients. The data from the 25 patients with seizures in our ICU dataset were used to evaluate the seizure detection performance using the fixed baseline feature normalization method (Ray et al., 2015).

**Figure 4:**
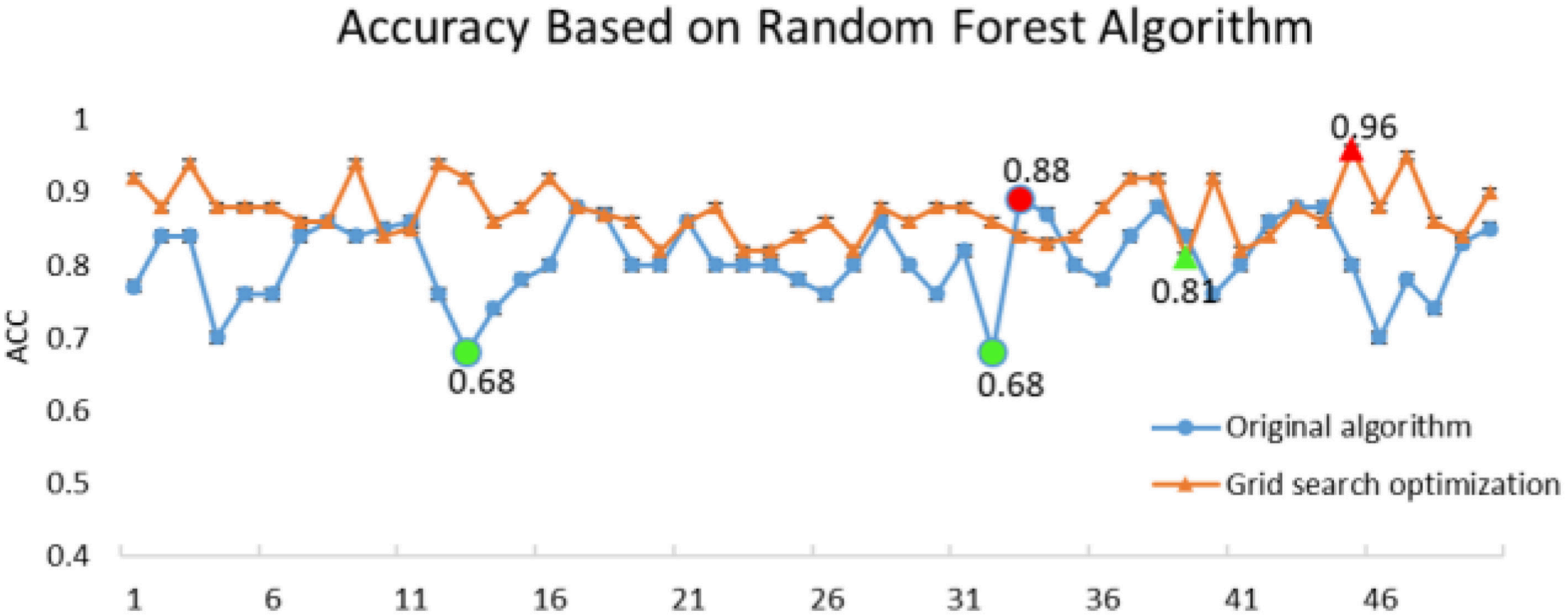
Comparison of execution accuracy between RF and RF-GSO.

This method is defined as shown in Equation.

(11) $$\begin{array}{l} ACC=\frac{TP+TN}{TN+FP+TP+FN}\cdot 100 \end{array}$$

where the true positive (TP) rate signifies the total number of true normal events recognized correctly, the true negative (TN) rate denotes the total number of true events of epileptic seizure period correctly identified, and the false positive (FP) and false negative (FN) rates are respectively the total number of false normal events and false events of epileptic seizure period incorrectly identified by the neuro-electrophysiologist or physician (Ktonas and Ventouras, 2014). The accuracy for correct classifications is shown in Figure 4.

**Figure 5:**
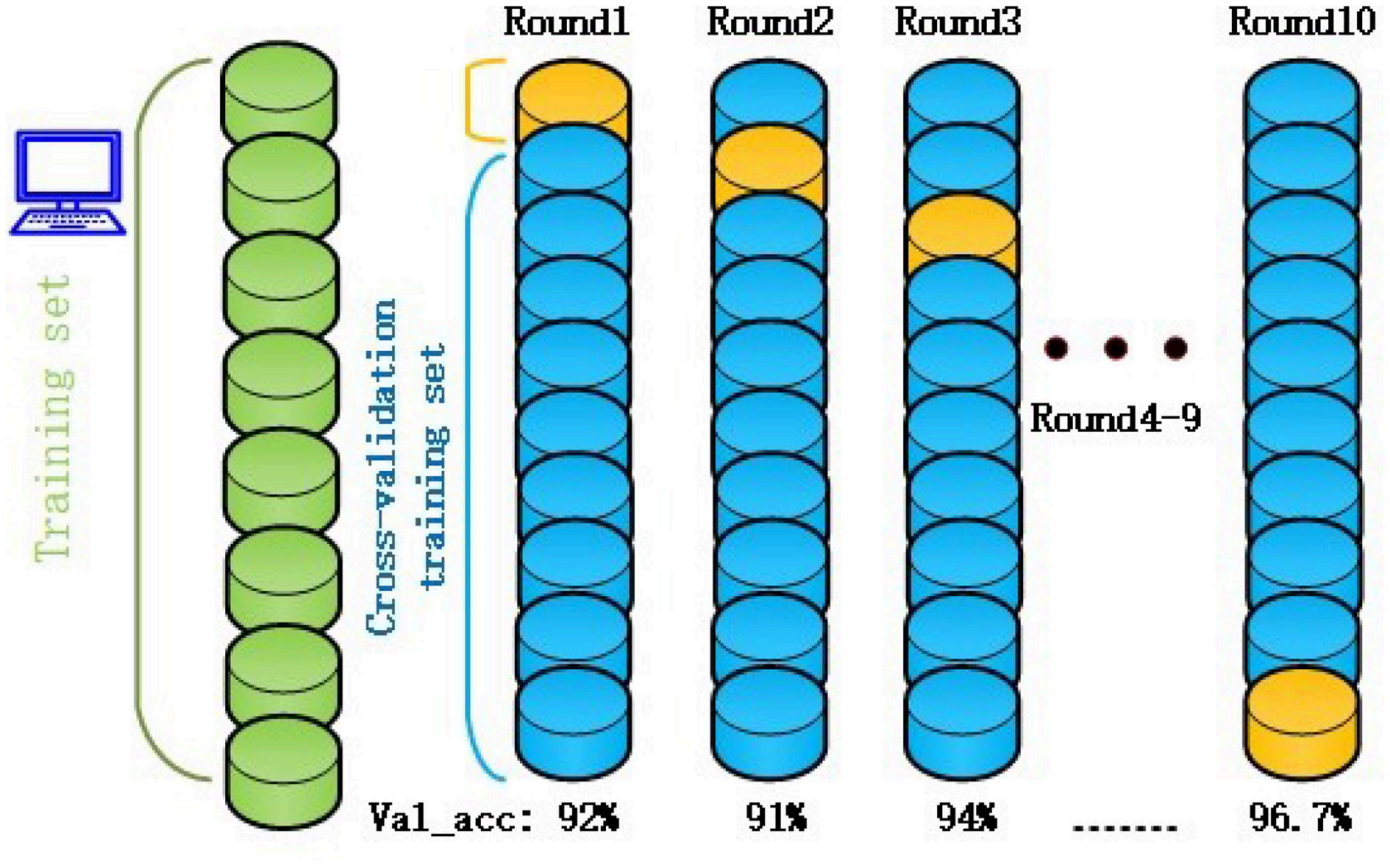
The accuracy of RF-GSO model under 10-fold CV.

The original RF algorithm of machine learning achieved a best performance of 88% for the 3-class. In summary, the penalty parameters min_sample_leaf, max_features, and n_estimators were the key parameters affecting the performance of the RF classifier. Therefore, the grid search algorithm divides the parameters to be searched into a grid with a certain spatial range, and searches the optimal parameters by traversing all the points in the grid to obtain the global optimal solution. The improved GSO not only increases the ACC of the RF model from 88 to 96.7%, but also increases the operating speed of the model. The success of the proposed model increased by nearly 10% points compared with the RF algorithm alone. Simultaneously, the RF-GSO model is trained using 10-fold CV, and its accuracy is shown in Figure 5. We can observe that the ACC of the training model is gradually increasing.

Related studies on automatic epilepsy detection systems are listed in Table 3. Most of the studies divided Bonn dataset into two-class: they can only distinguish between healthy subjects {OZ} and seizure-free intervals {FN}, seizure-free intervals {FN}, and seizure activity{S}, healthy subjects {OZ}, and seizure activity {S} for three time. This classification is so complicated for neuroscientists to re-manually control one by one. We adopted a combination of RF-GSO classification algorithm for machine learning to achieve high accuracy for the three-class, differentiating healthy subjects {OZ}, seizure-free intervals {FN}, and seizure activity {S} with a high ACC of 96.7% for one time. Simutanously, the excellent ACC is higher than Shafiul Alam's 80% (Alam and Bhuiyan, 2013) and Yuanfa Wang 93.9% (Wang et al., 2018) of bold values in Table 3. The integrative processing method reduces the complicated operation mode two types of classifications in the detection system. It should help neurologists to make clinic diagnosis decisions more conveniently and quickly.

### Performance Evaluation of Classification Model

After building the classification model, we tried to validate the model with more evaluation indicators of machine learning, not limited to the accuracy of the model. Furthermore, the model can be adjusted so that the model can achieve higher accuracy. Evaluation indicators mainly include a confusion matrix, a receiver operating characteristic, and an area under the curve. These indicators are deeper analysis of Alam and Bhuiyan (2013) the performance of a classification model from the perspective of classification errors, which is more important in medical diagnosis detection.

**Figure 6:**
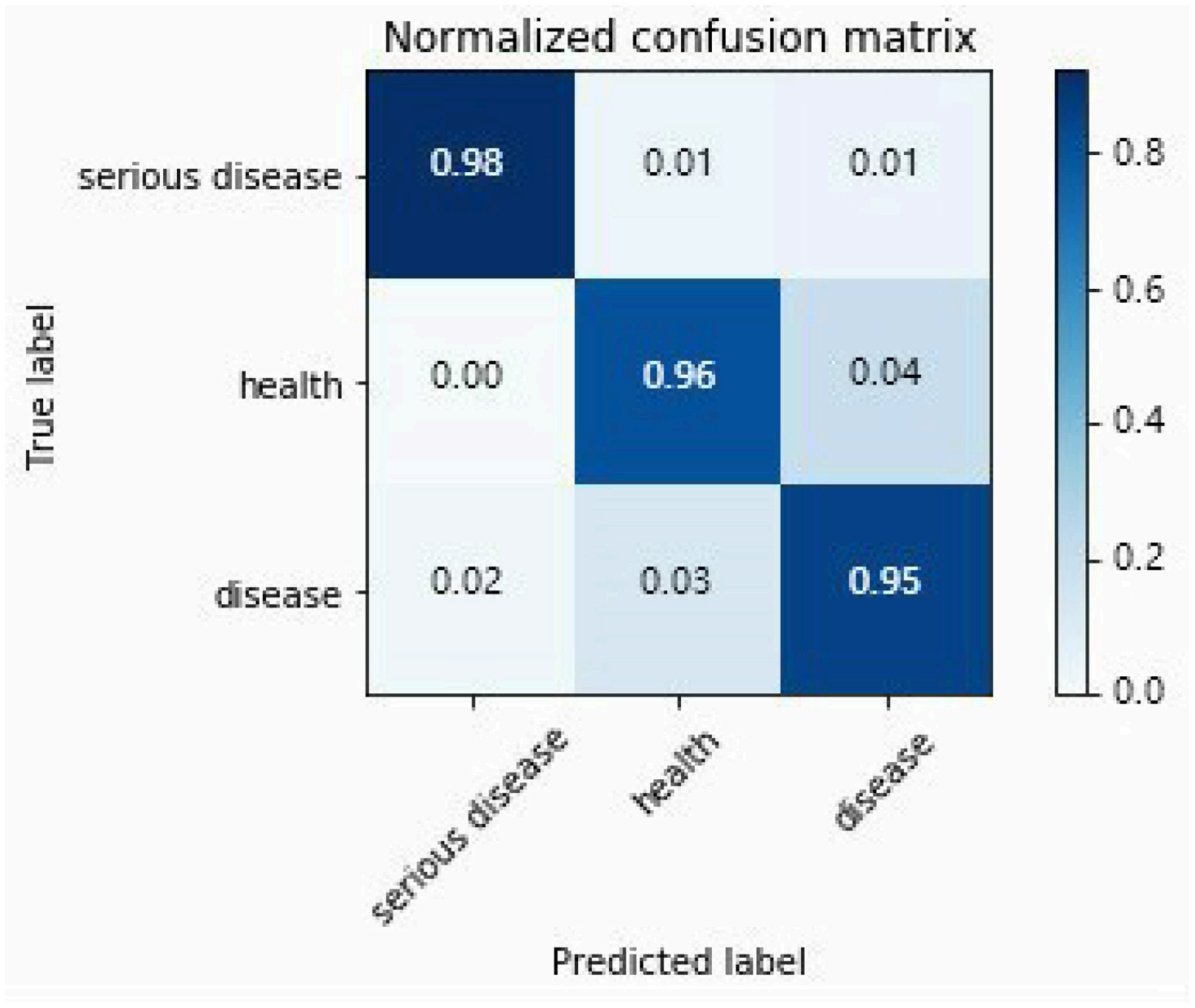
Three types classification confusion matrix.

A confusion matrix for assessing the three-category diagnostic classification problem of decision-making in epilepsy EEG is presented in Figure 6.

Horizontal and vertical direction of the confusion matrix are real targets and prediction results, respectively. True probability of serious epilepsy 98% as seen from the Figure 6. The fault classification mainly includes two state. The first state is the probability that the serious disease of S dataset is divided into O/Z datasets by 1%. The second state is the probability that the serious disease of S dataset is divided into F/N datasets by 1%. The classified probability of other types EEG can be similarly derived.

For different neurologists to make a diagnosis, they will have different considerations, which produce different confusion matrices. It is very difficult to evaluate a model by choosing a useful one from many confusion matrices.

This paper draws out different confusion matrices in a visual way. This makes it possible to objectively evaluate the classification effect of the classification model. The receiver operating characteristic (ROC) (Plöchl et al., 2012) curves of each EEG registration were obtained by drawing all possible true positive and false positive rates for the detection threshold, which are defined as shown in the following Equations (12, 13).

(12) $$\begin{array}{l} TPR=\frac{TP}{TP+FN}\cdot 100 \end{array}$$

False positive rate: this evaluation indicator is used to reject false detections of ictal.

(13) $$\begin{array}{l} FPR=\frac{FP}{TN+FP}\cdot 100 \end{array}$$

**Figure 7:**
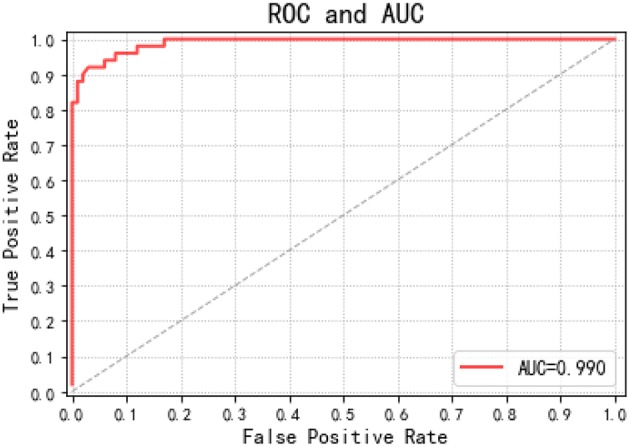
ROC curve and AUC value.

To allow a ROC curve to be drawn, the classifier must provide a confidence value that is judged as positive or negative for each sample. The ROC curve is drawn in Figure 7. If the ROC curve falls above the diagonal indicate the classification model has predictive ability, and conversely, there is no predictive ability. The ideal situation is that the ROC curve coincides with the y-axis, that is, the prediction ability is 100%. The proposed RF-GSO classification model has excellent classification performance as shown in Fig 10. The area under the curve (AUC) (Plöchl et al., 2012), which defines the average performance of the value classifier, is also used as a measure of the performance. In general, the AUC value ranges between 0.5 for a random performance to 1 for accurate classification. The AUC value of each patient is calculated, and the final performance measurement value is obtained as the mean of these 25 AUC values. Figure 6 shows an AUC 99.0% with the RF-GSO classification, indicating near perfect performance.

## Conclusion

The use of cEEG monitoring has changed the standard of care in the ICU; however, the long-term testing and monitoring of epilepsy patients is time consuming and laborious, and doctor time is limited. Therefore, we developed a novel RF-GSO automatic seizure detection of machine learning technique based on time–frequency analysis and PCA analysis. The experimental data demonstrate that it is very suitable for the classification task of epilepsy cEEG, especially into three or more types. However, the proposed novel model using RF with a GSO optimizer also has its limitations. If the noise in the EEG signals is too high, it will affect the detection of epilepsy. In practical clinical applications, it is essential to pre-process the EEG to eliminate various artifacts and noise before using the model. Our automatic detection framework of using RF algorithms and a GSO optimizer can auxiliary clinical diagnosis to detect epileptic episodes and make decisions more quickly, accurately, and effectively.

In the future, we intend to further optimize our model to achieve the classification and recognition of multiple-levels of epileptic seizure. It should also be applicable to other medical investigations, such as sport science application (Stone et al., 2018), detection of disorders of consciousness (Risetti et al., 2013), modulation of brain activity (Lapenta et al., 2013) brain computer interface (Li et al., 2010; Wang et al., 2016), and detection of EEG generated by different styles of music (Yan et al., 2014)assisting neurologists in their neuroscience field.

## Author Contributions

XW, GG, NL, and SQ conceptualized the study, reviewed and edited the writing. GG and NL contributed to the funding acquisition, the supervision, the validation of the study, and wrote the original draft. XW contributed to the data curation, the formal analysis, the investigation, the methodology, and the software.

### Conflict of Interest Statement

The authors declare that the research was conducted in the absence of any commercial or financial relationships that could be construed as a potential conflict of interest.
